# Distinction between Enterococcus faecium and Enterococcus lactis by a *gluP* PCR-Based Assay for Accurate Identification and Diagnostics

**DOI:** 10.1128/spectrum.03268-22

**Published:** 2022-12-01

**Authors:** Mireya Viviana Belloso Daza, Ana C. Almeida-Santos, Carla Novais, Antónia Read, Valquíria Alves, Pier Sandro Cocconcelli, Ana R. Freitas, Luísa Peixe

**Affiliations:** a Department for Sustainable Food Process, Università Cattolica del Sacro Cuore, Piacenza, Italy; b UCIBIO/REQUIMTE, Applied Molecular Biosciences Unit, Department of Biological Sciences, Laboratory of Microbiology, Faculty of Pharmacy, University of Porto, Porto, Portugal; c Associate Laboratory, Institute for Health and Bioeconomy, Faculty of Pharmacy, University of Porto, Porto, Portugal; d Clinical Pathology Service-Microbiology, Pedro Hispano Hospital, Matosinhos, Portugal; e TOXRUN – Unidade de Investigação em Toxicologia, Instituto Universitário de Ciências da Saúde, CESPU, CRL, Gandra, Portugal; University of Florida College of Dentistry

**Keywords:** *Enterococcus faecium*, *Enterococcus lactis*, PCR-based differentiation, *gluP*, public health

## Abstract

It was recently proposed that Enterococcus faecium colonizing the human gut (previous clade B) actually corresponds to Enterococcus lactis. Our goals were to develop a PCR assay to rapidly differentiate these species and to discuss the main phenotypic and genotypic differences from a clinical perspective. The pan-genome of 512 genomes of E. faecium and E. lactis strains was analyzed to assess diversity in genes between the two species. Sequences were aligned to find the best candidate gene for designing species-specific primers, and their accuracy was tested with a collection of 382 enterococci. E. lactis isolates from clinical origins were further characterized by whole-genome sequencing (Illumina). Pan-genome analysis resulted in 12 gene variants, with gene *gluP* (rhomboid protease) being selected as the candidate for species differentiation. The nucleotide sequence of *gluP* diverged by 90 to 92% between sets, which allowed species identification through PCR with 100% specificity and no cross-reactivity. E. lactis strains were greatly pan-susceptible and not host specific. Hospital E. lactis isolates were susceptible to clinically relevant antibiotics, lacked infection-associated virulence markers, and were associated with patients presenting risk factors for enhanced bacterial translocation. Here, we propose a PCR-based assay using *gluP* for easy routine differentiation between E. faecium and E. lactis that could be implemented in different public health contexts. We further suggest that E. lactis, a dominant human gut species, can cross the gut barrier in severely ill, immunodeficient, and surgical patients. Knowing that bacterial translocation may be a sepsis promoter, the relevance of infections caused by E. lactis strains, even if they are pan-susceptible, should be explored.

**IMPORTANCE** Enterococcus faecium is a WHO priority pathogen that causes severe and hard-to-treat human infections. It was recently proposed that E. faecium colonizing the human gut (previous clade B) actually corresponds to Enterococcus lactis; therefore, some of the human infections occurring globally are being misidentified. In this work, we developed a PCR-based rapid identification method for the differentiation of E. faecium and E. lactis and discussed the main phenotypic and genotypic differences of these species from a clinical perspective. We identified the *gluP* gene as the best candidate, based on the phylogenomic analysis of 512 published pan-genomes, and validated the PCR assay with a comprehensive collection of 382 enterococci obtained from different sources. Further detailed analysis of clinical E. lactis strains showed that they are highly susceptible to antibiotics and lack the typical virulence markers of E. faecium but are able to cause severe human infections in immunosuppressed patients, possibly in part due to gut barrier translocation.

## INTRODUCTION

Enterococcus faecium has emerged as a leading nosocomial multidrug-resistant (MDR) pathogen that is responsible for hospital-acquired infections worldwide (1). The population structure of E. faecium has been divided into distinct clades, with clade A mainly consisting of hospital- and animal-associated isolates and clade B containing community-associated isolates (2). In a previous study, we demonstrated that Enterococcus lactis and E. faecium from clade B are genetically and evolutionarily distinct from clade A E. faecium (3). In addition, other features distinguishing clade A from clade B E. faecium isolates included the common resistance of the former to different antibiotics (e.g., high levels of aminoglycosides, ampicillin, and/or vancomycin) and the enrichment of a variety of virulence factors and/or mobile genetic elements (3). Based on these data, it was proposed to reclassify clade B E. faecium as E. lactis because they are in fact the same species (3).

Although the extent of E. lactis strains causing human infections is much lower than that of clade A E. faecium, the strains are currently being misidentified as E. faecium in hospitals worldwide. Large epidemiological studies previously showed E. lactis genomes in association with a significant number of bacteremia isolates, as well as with vancomycin resistance (4). Also, many probiotics or feed formulas contain E. faecium, which might actually correspond to E. lactis (5). In this context, it is urgent to be able to easily differentiate between E. faecium and E. lactis, not only for accurate patient diagnosis and infection prognosis but also for correct taxonomic classification in different epidemiological and surveillance programs, in addition to industry purposes. Therefore, we developed a PCR assay for rapid detection of and differentiation between these species. Given the lack of studies characterizing E. lactis from hospitalized patients, we also explored the genomic and phenotypic features of the clinical E. lactis strains identified in this study.

## RESULTS

**FIG 1:**
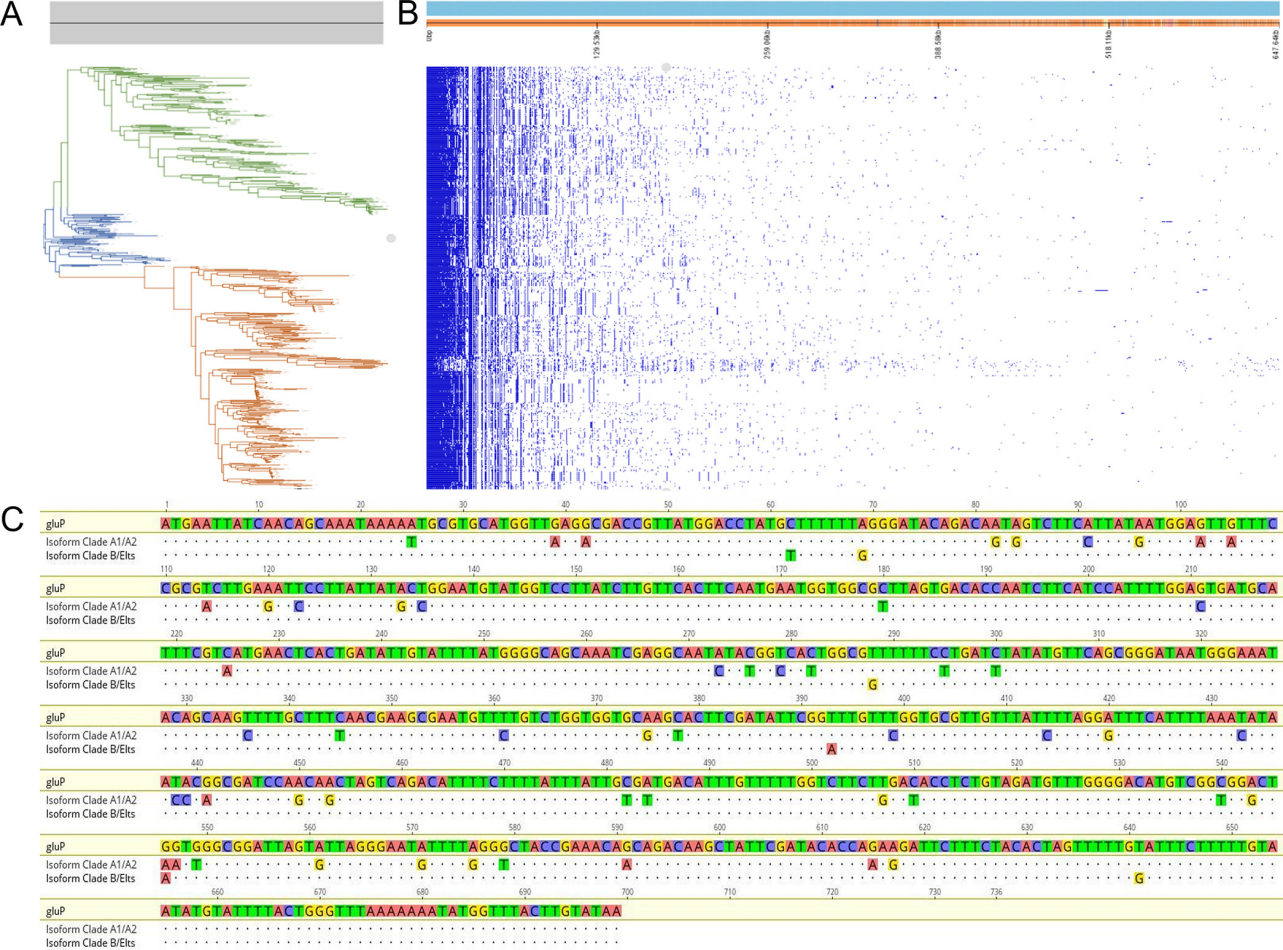
(A) Maximum likelihood phylogenetic tree of E. faecium and E. lactis based on core genome alignment, representing a clear clade separation of clade A1 E. faecium (red), clade-A2 E. faecium (blue), and clade B E. faecium/E. lactis (green). (B) Presence and absence matrix of the core and accessory genomes with respect to their phylogenetic positions. (C) Alignment of the *gluP* gene, showing the two main gene sequences from clade A/E. faecium (GenBank accession number UDP42194.1) and E. lactis (GenBank accession number WP_156271834.1), with different nucleotide patterns. Elts, E. lactis.

The first part of this study was to determine the pan-genome of 512 genomes, including 269 E. lactis genomes (with 183 genomes deposited as E. faecium and classified as clade B and 86 genomes deposited and classified as E. lactis) and 243 E. faecium genomes classified as clade A, as computed by digital DNA-DNA hybridization (dDDH) (Fig. 1A and Table S1). The pan-genome was composed of 32,380 genes, with 2% representing the soft core and core genome, which is defined as genes present in 95 to 100% of the genomes. The remaining 98% represented the accessory genome, which is defined by shell and cloud genes (>95% of the genomes) (Fig. 1B). The pan-genome analysis of the 512 genomes resulted in 12 genes with high enough variance between E. faecium and E. lactis (Table 1), of which 7 did not have a functional annotation. The remaining 5 genes with functional annotations included *araR* (arabinose transcriptional repressor), *gluP* (rhomboid protease), *rlmA* (23S rRNA [G745-N1]-methyltransferase), *ypjD* (inner membrane protein), and *yqgN* (inner membrane protein). The alignment of genes *araR_2*, *ypjD*, and *yqgN* did not show promising results, because they had high allelic variability among genomes from the same set. For genes *gluP* and *rlmA*, the alignment exhibited clear patterns of allelic differences between set 1 and set 2 of isolates. Nevertheless, the latter gene was not further explored for a ribosomal subunit-based PCR, mainly due to the presence of multiple copies that may introduce high variability and inaccuracy to the assay. Consequently, *gluP* was chosen for primer design and further screening analysis.

**TABLE 1:** Candidates for gene variants between E. faecium and E. lactis for primer design

Gene	Product	Function
*araR*	Arabinose metabolism transcriptional repressor	Transcriptional repressor of arabinose utilization genes
*comEA*	Hypothetical protein	
*gluP*	Rhomboid protease GluP	Rhomboid-type serine protease that catalyzes intramembrane proteolysis; important for normal cell division and sporulation
Group_12706	Hypothetical protein	
Group_16273	Hypothetical protein	
*rimI*	Hypothetical protein	
Group_21758	Hypothetical protein	
Group_21783	Hypothetical protein	
Group_21801	Hypothetical protein	
*rlmA*	23S rRNA (guanine745-N1)-methyltransferase	Methylation of 23S rRNA nucleotide G745
*ypjD*	Putative protein YpjD	Inner membrane protein YpjD
*yqgN*	Putative protein YqgN	Uncharacterized protein YpjD

The alignment of *gluP* from all E. faecium and E. lactis genomes showed two different nucleotide sequences; set 1/set 1 and set 2/set 2 identities ranged from 98 to 100%, and set 1/set 2 identities ranged between 90 and 92% (Fig. 1C; also see Table S2 in the supplemental material). Once the primers were designed for each species-specific sequence (sequences and PCR conditions are presented in Table 2), we submitted them to BLAST to evaluate their *in silico* accuracy with all deposited genomes in the NCBI database. Indeed, the primer pair for clade A showed 100% identity with the corresponding sequences of clade A isolates and <90% identity with E. lactis *gluP* sequences. Similar results were obtained in the analysis of E. lactis primers*. In silico* PCR of both primers resulted in 100% specificity with E. faecium ATCC 700221 *and* E. lactis LMG 25958 type strains.

**TABLE 2:** Species-specific primers and PCR conditions for differentiation of E. faecium and E. lactis species by amplifying the *gluP* gene

Species and primer type	Sequence (5′ to 3′)	Length (bp)	Start position	Stop position	*T_m_* (°C)	GC content (%)	Product size (bp)	PCR conditions
E. faecium								
Forward	GCGTGCATGGTTAAGACGAC	20	27	46	59.91	55	427	1 cycle of 10 min at 94°C; 30 cycles of 30 min at 94°C, 30 min at 61°C, and 30 min at 72°C; and 1 cycle of 10 min at 72°C
Reverse	CTGCTGGATCGCTGGGTTAT	20	453	434	59.89	55		
E. lactis								
Forward	TACGGTCACTGGCGGTTTTT	20	274	293	60.18	50	324	1 cycle of 10 min at 94°C; 30 cycles of 30 min at 94°C, 30 min at 58°C, and 30 min at 72°C; and 1 cycle of 10 min at 72°C
Reverse	TGTCTGCTGTTTCGGTAGCC	20	597	578	60.32	55		

We then tested and validated the PCR assay with enterococcal collections recovered by the group in different surveillance studies over diverse time periods. The PCR assay showed 100% accuracy when testing the 137 well-characterized E. faecium isolates, with clade A isolates being amplified with primers A1/A2 and E. lactis (former clade B E. faecium) isolates being amplified with primers B/E. lactis exclusively (see Table S3). The 27 E. lactis strains identified were obtained from human colonization (*n* = 17), human clinical settings (*n* = 5), animals (*n* = 3), and the environment (*n* = 2) (see Table S3). At least in our data set, E. lactis isolates were generally more susceptible to antibiotics than E. faecium isolates (see Table S3); only 1 E. lactis isolate was resistant to ampicillin (hospital sewage), 2 were resistant to ciprofloxacin, and all the rest expressed resistance to erythromycin, tetracycline, and/or aminoglycosides. We used the multilocus sequence typing (MLST) E. faecium scheme to provide an overview of E. lactis clonal diversity and confirm their assignment as typical clade B E. faecium clones; they belonged to 18 different sequence types (STs) (4 novel), some previously associated with human clinical settings (ST74, ST108, ST123, ST329, ST361, ST798, and ST994), hospital surveillance and environment (ST123, ST361, and ST717), community settings (ST118 and ST798), and animals (ST75 and ST798) in different countries (https://pubmlst.org/bigsdb?db=pubmlst_efaecium_isolates). Among the 245 isolates, amplifications were also highly specific; 98 isolates (from human colonization) were amplified only with B/E. lactis primers, 101 (50 from human clinical settings and 51 from human colonization) were amplified only with clade A E. faecium primers, and the remaining (*n* = 46) were negative for both primer pairs and then confirmed as E. faecalis. Most (102/180 isolates [57%]) E. lactis strains identified among all 382 enterococci (245 plus 137 isolates) originated from human fecal colonization.

**FIG 2:**
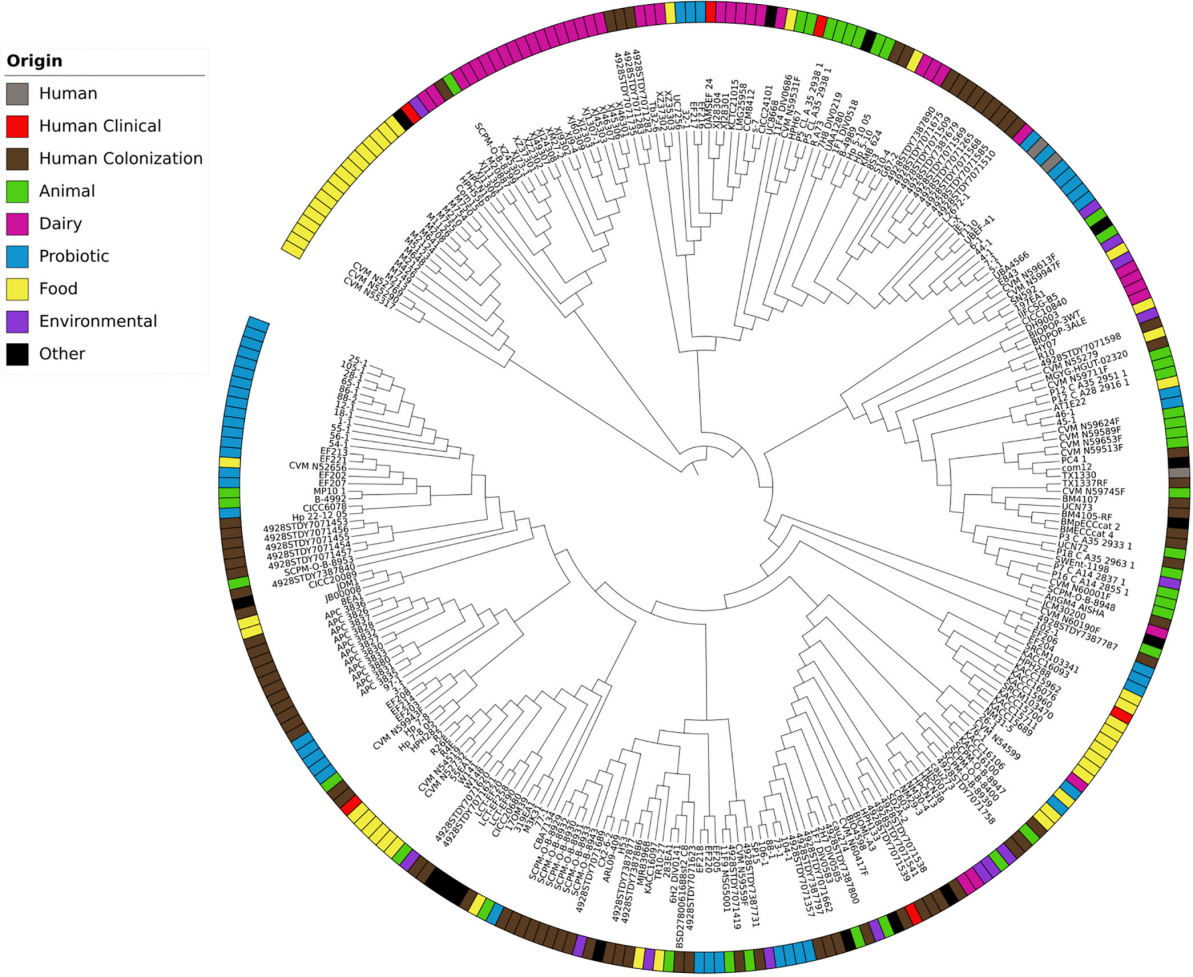
Maximum likelihood phylogenetic tree of 274 E. lactis genomes based on core genome alignment. Different isolation origins are classified as follows: human clinical samples in red, human colonization samples (stool, gastrointestinal, genitourinary, and breast milk samples) in brown, human samples of undetermined origin in gray, animal isolates in green, food samples in yellow, dairy samples in magenta, probiotic samples in blue, environmental samples in purple, and samples with other origins (unknown) in black. No clear patterns among isolates from different sources are visible. Clinical E. lactis samples are clustered among probiotic/dairy and animal samples.

Details from the 5 clinical E. lactis strains identified in this study (2 from blood samples, 2 from bile samples, and 1 from an abdominal pus sample) are included in Table 3. All patients but 1 presented comorbidities and cholangitis/cholecystitis pathologies, for which gut bacterial translocation has been proposed as a possible cause. Indeed, these patients were coinfected with Gram-negative bacteria in 3/5 cases and underwent broad-spectrum antibiotic therapy, which is known to favor enterococcal overgrowth in the lumen and possible gut translocation (6). Three of the 5 isolates expressed resistance to erythromycin only, and the remaining were pan-susceptible. Although 3 of them carried some *pbp5* amino acid mutations and the ResFinder 4.1-predicted phenotype was one of resistance in those cases, all presented a sensitive phenotype against ampicillin (MICs of 0.05 to 0.75 mg/L). This may be explained by the absence of key mutations that are frequent among clinical ampicillin-resistant E. faecium strains (7). Regarding antibiotic resistance genes, only *aac(6′)-Ii* and *msr(C)* genes were found, although both are intrinsic for E. faecium and should be for E. lactis as well. Actually, we submitted both genes to BLAST against all E. lactis genomes, and they were present in 100% of them. Although the MLST scheme was designed for E. faecium and not for E. lactis, the 5 isolates were identified as ST118, ST329, ST361, ST994, and ST2215, and all but ST118 and the last one, which is novel, have been identified previously among hospitalized patients (https://pubmlst.org/bigsdb?db=pubmlst_efaecium_isolates) (4). These 5 E. lactis genomes were further compared with available E. lactis genomes (*n* = 269), and the resulting phylogenetic tree with the 274 E. lactis genomes clearly shows the intermixing of E. lactis genomes from different sources, with no obvious separation of isolates by source (Fig. 2). The 5 clinical E. lactis strains clustered with probiotic, dairy, and animal samples. Additionally, they carried *acm*, *sgrA*, *ccpA*, *bepA*, *gls*, and *pil* genes, which are involved in different cellular functions (see Table S4), but most of them (59%) either were truncated (32%) or presented low levels of similarity (27%) to reference E. faecium strains.

**TABLE 3:** Epidemiological data and characterization of clinical E. lactis isolates from a Portuguese hospital in the Porto area

Isolate^*d*^	ST^*a*^	Sex/age (yr)^*b*^	Date of isolation (day/mo/yr)	Sample type	Pathology	Hospital unit	Clinical case^*c*^	Coexisting bacteria	Antibiotic therapy	Comorbilities
CCP212	2215	M/63	11/10/19	Bile	Cholangitis	Surgery	Hospitalization because of episode of cholangitis; antibiotic therapy with Pip and Taz and percutaneous transhepatic cholecystectomy with bile aspiration; bile culture results: ESBL-positive E. coli sensitive to gentamicin and ertapenem and E. faecium; treatment with meropenem	ESBL-positive E. coli sensitive to gentamicin and ertapenem	Pip plus Taz and meropenem	Chronic pancreatitis of alcoholic etiology with multiple episodes of cholangitis
CCP213	329	M/70	28/10/19	Abdominal pus	Necrotizing fasciitis	Medicine	Previous hospitalization for septic shock with abdominal necrotizing fasciitis after elective cholecystectomy; surgery with pus collection	E. coli resistant to amoxicillin and cefuroxime and P. aeruginosa	Pip plus Taz and vancomycin, with addition of clindamycin	Ischemic heart disease since 1995, chronic obstructive pulmonary disease, CKD, and gallstones
CCP214	361	M/80	28/09/20	Bile	Cholecystitis	Surgery	Laparoscopic cholecystectomy due to acute lithiasic cholecystitis, initiation of Pip plus Taz treatment, collection of bile during surgery, isolation of cefotaxime-sensitive and Pip- and Taz-resistant E. coli and ampicillin-sensitive E. faecium, and switch to cefotaxime plus ampicillin; discharged on 5 October 2020.	E. coli resistant to Pip and Taz	Pip plus Taz	Nonrelevant
CCP215	994	M/84	25/02/22	Blood	Cholangitis	Medicine	Multiple complications associated with left total hip prosthesis with prolonged hospital stay for periprosthetic infection with prosthesis extraction, grade II acute cholangitis and prerenal AKI superimposed on CKD, and initiation of antibiotic therapy with Pip plus Taz; hemoculture results: E. faecium; excellent clinical evolution, with good response to antibiotic therapy, having completed 14 days of Pip plus Taz treatment	None	Pip plus Taz	Arterial hypertension, obesity, diabetes mellitus (type II), and CKD
CCP216	118	F/64	23/05/22	Blood	Cholangitis	Surgery	During hospitalization, maintained controlled pain; progressive decrease in inflammatory parameters without leukocytosis, decrease in cholestasis parameters, and progressive decrease in lipase and amylase levels, thus currently no criteria for ERCP; at discharge, with innocent abdominal palpation, significant improvement in jaundice and in sustained apyrexia	None	Ceftriaxone plus metronidazole	Arterial hypertension, gastroesophageal reflux disease, dyslipidemia, depressive/anxious disorder, lower limb venous insufficiency, and colonic diverticulosis

a ST was defined according to the MLST scheme for E. faecium because there is no scheme for E. lactis.

b M, male; F, female.

c Pip, piperacillin; Taz, tazobactam; ESBL, extended-spectrum β-lactamase; AKI, acute kidney injury; CKD, chronic kidney disease; ERCP, endoscopic retrograde cholangiopancreatography.

d Isolates CCP212, CCP213, CCP214, CCP215 and CCP216 correspond to HPH55b, HPH67, HPH133, HPH282 and HPH288, respectively, the name appearing in the GenBank project.

## DISCUSSION

The emergence of MDR E. faecium strains in hospitals, causing tenacious and hard-to-treat infections, over past decades has been alarming and has intensified the need to distinguish strains of public health concern. Currently, some of the enterococci causing hospital infections are being misidentified worldwide. In this work, we corroborate a recent proposal that a subset of E. faecium (clade B) strains are actually E. lactis (3), and we designed primers to correctly differentiate between these species for accurate identification. The primer design was based on the pan-genome alignment of the two species, aiming to find unique genes or gene variations that were sufficiently discriminatory to differentiate them through standard PCR. The *gluP* gene, coding for a rhomboid protease, showed two different sequences with enough nucleotide pattern differences to design species-specific primers. Rhomboid family proteases are a ubiquitous family of intramembrane serine proteases, with a unique evolutionary conservation level (8). Different studies have been conducted to investigate the structure and function of rhomboid proteases, especially AarA in Providencia stuartii (a role in quorum sensing), GlpG in Escherichia coli and Haemophilus influenzae (a role in antibiotic susceptibility), and GluP (also called YqgQ) in Bacillus subtilis (a role in cell division and glucose uptake) (9). The function and structure descriptions of GluP within the *Bacillota* phylum may suggest the potential function of GluP in enterococci; however, this exceeds the scope of this study (10). More research will unveil the phenotypic impact of the allelic differences between species, but for the scope of this study only the genotypic variation was considered.

E. lactis is genomically and evolutionarily distinct from E. faecium. Phenotypically, the strains are generally much more susceptible to antibiotics and lack key virulence markers known to be associated with outbreak/epidemic E. faecium strains (this study and reference 3). Although E. lactis strains seem less prone to cause human infections, clade B E. faecium strains have been described as being able to acquire the VanA (4) or VanN operon (11), and their proportions among human infections caused by enterococci may be undervalued since most surveillance studies focus on MDR E. faecium strains. According to the features of the clinical E. lactis strains detected in this and other (previously described as clade B E. faecium) studies and their great association with human fecal colonization, we think that E. lactis, as one dominant human gut species, can cross the gut barrier in severely ill, immunodeficient, and/or surgical patients. Indeed, all patients infected by E. lactis in this study presented at least one risk factor for bacterial translocation (chronic diseases such as pancreatitis, abdominal surgeries, or broad-spectrum antibiotics). Because the ability of different enterococcal species to translocate into host tissues seems evolutionarily related (12), it makes sense that E. lactis is able to do it as well. Previous studies showed that enterococci were enriched in the fecal microbiome of patients with sclerosing cholangitis, together with Gram-negative bacteria (13), and that is one of the most common genera in bile cultures (14); therefore, more research is needed to determine the amount of E. lactis versus other enterococcal species in these and other clinical cases. In common with previous studies describing E. lactis in association with bloodstream infections (4, 15, 16), here we describe two E. lactis bacteremia cases with clinical significance and systemic signals of infection. One limitation of our study is the small sample size, but future large-scale studies will determine the real ability of E. lactis to cause bacteremia and other infections, as well as the best antibiotic therapy to treat them.

To conclude, we designed and validated a PCR assay to discriminate between E. faecium and E. lactis species. We note that published primers that have been widely used for years and designed to identify E. faecium (e.g., *ddl* gene specific) lack enough discriminatory power to distinguish these species. Very recent approaches to differentiate the species by matrix-assisted laser desorption ionization–time of flight mass spectrometry (MALDI-TOF MS) or quantitative PCR (qPCR) showed promising results (17, 18), but until we have a robust collection of E. lactis mass spectra for routine hospital identification and other purposes, we have successfully designed a highly specific PCR that can be applied in a cost-effective and timely fashion. The development of a precise differentiation method has direct implications in both the clinical and food safety fields and could identify E. faecium strains currently being used in probiotics and feed that actually correspond to E. lactis and/or strains associated with human infections that are actually E. lactis, with possible implications for infection management and overall in different public health contexts.

## MATERIALS AND METHODS

### Pan-genome analysis and species-specific primer design.

A total of 512 enterococcal genomes were retrieved from the NCBI GenBank database. The genomes were submitted to the genome-to-genome distance calculator online tool to compute dDDH values against E. faecium type strain ATCC 700221, to discriminate between clade A (≥70%) and clade B/E. lactis (<70%) (see Table S1 in the supplemental material) (19). At the time of analysis (January 2022), the selection of strains was based on the level of genome completeness (only complete assemblies), as well as their relevance in the clinical or agri-food fields, as measured by their inclusion in different publications (see Table S1). Genomes were annotated with Prokka (19) and submitted to pan-genome analysis using Roary v3.11.2 (20). The output discriminates core genes as being present in 95 to 100% of the strains of interest. The accessory genes are constituted by shell (15% to <95%) and cloud (0% to <15%) genes. To differentiate between the two species and create specific primers, we analyzed unique genes in E. lactis that could be absent in E. faecium or gene variants that differed between the two species by using the query_pan_genome command. We defined set 1 as containing E. lactis isolates and set 2 clade A E. faecium isolates. Gene variants were then extracted from all genomes of set 1 and were subsequently aligned to evaluate allelic differences among set 2 isolates. We also submitted these genes to BLAST (21) to corroborate the allelic variance between E. lactis and E. faecium genomes. All extraction and alignment steps were performed with Geneious Prime v2022.0.1. Good gene candidates were furtherly evaluated to test the accuracy of this method, and the selected gene was used for primer design with Primer3 (22). Finally, the proposed primers were tested *in silico* with the genomes of E. faecium ATCC 700221 and E. lactis LMG 25958 type strains and *in vitro* with a collection of 137 well-characterized E. faecium strains (61 from human clinical origins, 42 from animals, 21 from healthy individuals, and 13 from miscellaneous sources) that had been classified as clade A (*n* = 110) or clade B (*n* = 27) in previous surveillance studies (15). The primers were also tested with 245 enterococcal isolates for which identification and/or clonality was not established (unknown clade or even species), to test eventual cross-reactions between E. lactis, E. faecium, and non-E. faecium/E. lactis isolates. PCR was performed in a Bio-Rad iCycler system with PCR conditions and primer details as described in Table 2.

### Antibiotic susceptibility and genomic profiling of clinical E. lactis isolates.

A total of 5 clinical isolates identified as E. lactis with the designed primers were further analyzed and identified with the following strain names: CCP212, CCP213, CCP214, CCP215 and CCP216. The 5 strains were subjected to antimicrobial susceptibility testing, which was performed with disk diffusion assays against 12 antibiotics (ampicillin, vancomycin, teicoplanin, ciprofloxacin, erythromycin, gentamicin, streptomycin, linezolid, tigecycline, tetracycline, chloramphenicol, and quinupristin-dalfopristin). In general, we used EUCAST v12.0 criteria; in cases in which EUCAST did not specify a clinical breakpoint, we referred to CLSI guidelines (23). Additionally, ampicillin MICs were determined by Etest (Liofilchem) (7).

Genomic DNA was extracted from 1 mL of overnight cultures in brain heart infusion broth using a Wizard genomic DNA purification kit (Promega Corp., Madison, WI, USA) according to the manufacturer’s instructions, and the concentration was determined with a Qubit 3.0 fluorometer (Invitrogen, Thermo Fisher Scientific, Waltham, MA, USA). Genome sequencing was accomplished with an Illumina NovaSeq 6000 platform (2 × 300-bp pair-ended runs) (genome size, ~6 Gb; coverage, 100×) according to standard Illumina protocols, at Eurofins Scientific (Italy). Data were analyzed using FastQC (http://www.bioinformatics.babraham.ac.uk/projects/fastqc) to test the quality of the raw and preprocessed data, SPAdes v3.10.0 to perform *de novo* assembly of the paired-end reads, and QUAST (http://bioinf.spbau.ru/quast) to evaluate the quality of the genome assembly. After annotation with Prokka and pan-genome and core genome analyses with Roary, a maximum likelihood phylogenetic tree was constructed with the core genome alignment of the 269 E. lactis genomes (see Table S1) and the 5 clinical E. lactis genomes (this study) using RAxML v1.0 (24), and results were edited using iTOL (25).

### Data availability.

This Whole Genome Shotgun project including the 5 clinical E. lactis isolates has been deposited at DDBJ/ENA/GenBank under BioProject accession number PRJNA851953 under the accession JAMYDK000000000-JAMYDN000000000 and JANDLX000000000 numbers.
